# A systematic in-silico functional and structural analysis reveals deleterious missense nsSNPs in the human *CSF1R* gene

**DOI:** 10.22099/mbrc.2025.53206.2156

**Published:** 2025

**Authors:** Purvi Malhotra, Aaryan Jaitly, Harshil Walia, Ojasvi Dutta, Deepanshi Rajput, Mujtaba Husaini, Chander Jyoti Thakur, Sandeep Saini

**Affiliations:** 1 Department of Bioinformatics, Goswami Ganesh Dutta Sanatan Dharma College, Sector 32, Chandigarh, 160030, India; 2 Department of Biophysics, Panjab University, Sector-25, Chandigarh, 160014, India

**Keywords:** SNP, bioinformatics, conservation, domain analysis, mutation

## Abstract

Colony Stimulating Factor-1 Receptor (CSF1R) is a tyrosine kinase transmembrane receptor that plays a vital role in innate immunity and neurogenesis and controls the differentiation and maintenance of most tissue-resident macrophages. *CSF1R* mutations have been linked with many neurodegenerative diseases. In this work, we aim to identify the functional and structural impact of deleterious non-synonymous single nucleotide polymorphisms (nsSNPs) mutations on CSF1R, which could help understand the consequences of these mutational changes. A consensus-based prediction approach was used to screen the missense SNPs using six in-silico tools: SIFT, PROVEAN, PMut, MutPred, MISSENSE 3D, and FATHMM. SNPs found to be deleterious by more than five out of six tools were subjected to further analysis, such as protein secondary structure and domain architecture analysis by PSIPRED and NCBI-CDD, respectively. Mutant models of highly deleterious SNPs were modeled using PyMol, followed by energy minimization and Root Mean Square Deviation (RMSD) analysis and molecular dynamic (MD) simulation by YASARA, TM-ALIGN, and WebGro, respectively. Out of 780 missense SNPs screened, we found the four most deleterious SNPs (L301S, A770P, I775N, and F849S) that decreased the protein stability because of their presence in the conserved regions of wild-type CSF1R. Structural and functional studies revealed that these mutations could disrupt the protein's core and surface interactions, leading to destabilization and functional impairment. Moreover, the mutated proteins exhibited enhanced conformational flexibility and instability, as confirmed by MD simulation analysis.

## INTRODUCTION

Colony stimulating factor-1 receptor (CSF1R), belonging to the type III protein tyrosine kinase receptor family, is recognized as the cell surface receptor for the macrophage colony-stimulating factor 1 (CSF-1). The proto-oncogene c-fms, which is located on chromosome 5q33.3, is known to encode it [1]. CSF1R consists of extracellular domains, a transmembrane domain, and an intracellular tyrosine kinase domain [2]. CSF1R, known for its multifaceted roles, is considered to play a fundamental function in innate immunity by governing the proliferation of various cell types, including tissue macrophages, osteoclasts, Langerhans cells in the skin, Paneth cells in the small intestine, and microglia in the cerebrum. Predominantly expressed in microglia within the central nervous system, CSF1R is believed to be crucial for their proliferation and maintenance under normal conditions [3]. Moreover, its broad tissue expression pattern has been descibed as pivotal in various pathological conditions such as neoplastic, inflammatory, and neurological diseases [2].

The discovery and purification of CSF-1 led to the recognition of CSF1R and the demonstration of its intracellular tyrosine kinase domain activity [4-6]. Studies have shown the human CSF1R has been shown to share around 75% and 84% overall homology with the mouse and feline versions, respectively [7, 8]. Besides this, it has been demonstrated in several studies that genetic excision or loss of *CSF1R* function results in microglial depletion across species, highlighting the dependence of microglial proliferation and development on CSF1R [9, 10].

Genetic variations of the *CSF1R* gene have been implicated in several neurodegenerative diseases. For instance, loss-of-function mutations in the *CSF1R* gene are the major cause of adult-onset leukoencephalopathy with axonal spheroids and pigmented glia (ALSP) [11]. Single nucleotide polymorphisms (SNPs) associated with the *CSF1R* gene are associated with inhibitor development in hemophilia A [12]. Furthermore, these mutations have also been linked to hereditary diffuse leukoencephalopathy with spheroids (HDLS) and pigmented orthochromatic leukodystrophy (POLD) [13, 14].

SNPs are regarded as the most prevalent form of chromosomal variations, occurring once every 100–300 base pairs, and are known to have a crucial impact on disease outcomes [15, 16]. Among them, non-synonymous single nucleotide polymorphisms (nsSNPs) are particularly noted for their potential to alter the structure and function of the protein due to modifications in the amino acid sequence. Such modifications can significantly influence the disease’s development and progression [17]. 

Given the large amounts of variation data generated through genome sequencing efforts, it is considered challenging to study the effect of these mutations on gene function and their encoded proteins through experimental methoda alone [18]. Therefore, a large number of previous studies have been conducted by research community to screen these large variation datasets using in-silico approaches [19-28].

Considering the involvement of *CSF1R* mutations in several diseases and the extensive SNP dataset that may not be feasibly analysed through experimental approaches alone, this study was designed to screen the missense SNPs of the *CSF1R* gene and to investigate their damaging effects on protein stability using in-silico tools. Furthermore, the screened high-risk SNPs were analysed for their effects on domain architecture and for changes in the modeled mutant’s secondary and tertiary protein structures. 

## MATERIALS AND METHODS

### Retrieval of dataset:

**Figure 1:**
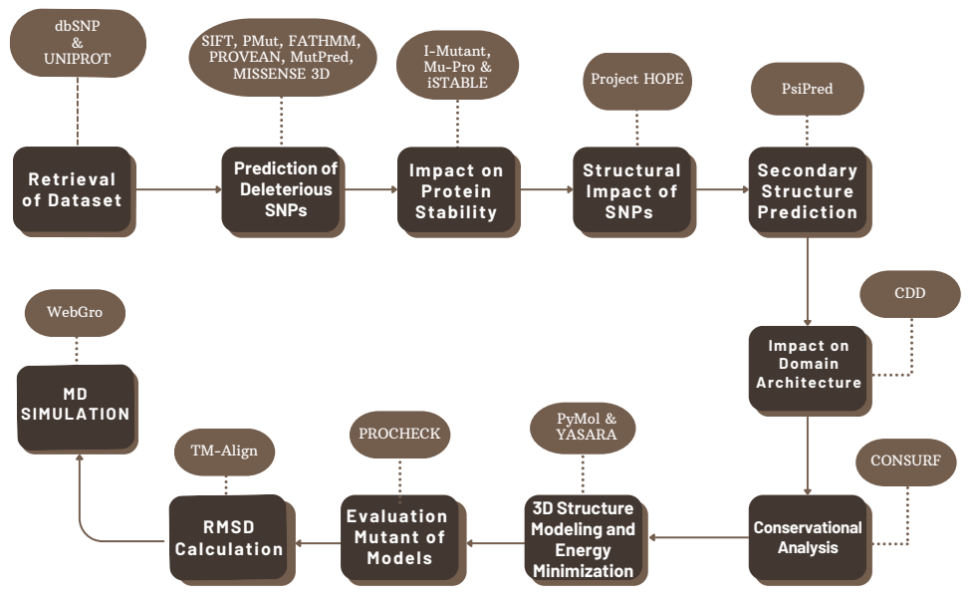
Flowchart of methodology showing the steps followed during analysis

 The SNP data for the *CSF1R* gene was retrieved from NCBI SNP database (dbSNP) (https://www.ncbi.nlm.nih.gov/snp) [29]. Only missense SNPs were selected, as they are potentially capable of altering protein structure and function. The sequence of the CSF1R protein (UniProt accession no. P07333) was also retrieved from the UniProt database (https://www.uniprot.org/) [30]. A summary of the steps followed in the study is provided in Fig. 1. 

### Prediction of deleterious nsSNPs:

 The missense SNPs retrieved from dbSNP were screened using six bioinformatics tools, namely, SIFT (Sorting Intolerant From Tolerant, https://sift.bii.a-star.edu.sg/) [31], PROVEAN (PROtein Variation Effect Analyzer, http:// provean.jcvi.org/index.php) [32], FATHMM (Functional Analysis Through Hidden Markov Model, http://fathmm.biocompute.org.uk/) [33], PMut (http://mmb.irbbarcelona.org/PMut/) [34], Missense3D-DB (http://missense3d.bc.ic.ac.uk:8080/) [35] and MutPred2 (http://mutpred. mutdb.org/) [36].

SIFT classifies nsSNPs as deleterious or neutral. The scoring system ranges from 0 to 1. An amino acid substitution is classified as deleterious if the score is <= 0.05, and tolerated if >0.05 [31]. Moreover, PROVEAN classifies single or multiple amino acid substitutions, in-frame insertions, and deletions as deleterious if the score is ≤ -2.5 and neutral if >-2.5 [32].

To predict the functional implications of missense mutations, FATHMM was employed, which integrates sequence conservation via Hidden Markov Models and “pathogenicity weights” to assess tolerance to mutations. Variants scoring below the threshold of 0.75 are identified as potentially cancer-associated [33]. Furthermore, PMut was used to classify mutations as disease-causing or neutral. Scores above 0.5 are considered disease-causing, while scores below indicate neutrality [34].

Moreover, Missense3D was utilized to assess structural effects of amino acid substitutions using 16 structural parameters, categorizing them as “Damaging” or “Neutral” [35]. Besides this, MutPred2 was applied to predict whether substitutions are pathogenic or benign, using a threshold score of 0.5. Substitutions scoring above 0.5 are interpreted as likely affecting protein function [36].

### Predicting the protein stability:

We used three web tools, namely, I-Mutant 2.0 (https://folding.biofold.org/cgi-bin/i-mutant2.0.cgi) [37], iSTABLE (http://predictor.nchu.edu. tw/iStable/) [38], and MuPro (http://mupro.proteomics.ics.uci.edu/) to predict the stability of mutant protein sequences [39]. I-Mutant 2.0 utilizes a support vector machine (SVM) algorithm to predict the effect of amino acid mutations on protein stability. It calculates the energy change** (**∆∆G) value by subtracting the unfolding Gibbs free energy of the wild type from the unfolding Gibbs free energy of the mutated protein [37]. Similarly, iSTABLE is an integrated predictor constructed using sequence information and prediction results from different predictors to provides output in the form of ∆∆G values [38].

Additionally, MuPro, a machine learning (ML) classifier was used to predict the effect of amino acid change on protein stability. It predicts ΔΔG and classifies mutations with a confidence score based on the effects on stability, using both SVM and neural networks [39].

### Analysis of structural impacts of point mutations:

 The project HOPE (Have (y)Our Protein Explained) (https://www3.cmbi.umcn.nl/hope/input/) server was used to analyze the structural effect of mutations on proteins and gain insights into the differences in properties between wild-type and mutant amino acids at specific positions. Information from both 2D and 3D structures, along with sequence annotation data from various protein structural and sequence analysis algorithms, is integrated to provide comprehensive predictions [40].

### Secondary structure prediction:

PSIPRED (http://bioinf.cs.ucl.ac.uk/psipred/) was utilized to predict the secondary structure of the mutant proteins. The FASTA sequences were submitted as input and resulting annotations were provided using color codes: helices in pink, strands in yellow, and transmembrane regions in grey [41]. 

### Analysis of domain architecture:

 The NCBI Conserved Domain Database (CDD) (https:// www.ncbi.nlm.nih.gov/Structure/cdd/wrpsb.cgi) was utilized to predict the impact of mutations on the domains of the CSF1R protein. CDD is based on reverse position-specific (RPS) BLAST, a variant of PSI-BLAST, to scan the query protein against a set of pre-calculated Position Specific Scoring Matrices (PSSM) [42, 43]. The wild-type UniProt ID and mutant FASTA sequences were used for the domain analysis.

### Conservational analysis:

 The ConSurf server (https://consurf.tau.ac.il/) was used to assess the evolutionary conservation scores for each residue using a Bayesian method. Conservation was visualized using a 1–9 scale, where 9 represents the most conserved residue and 1 indicates the least conserved [44]. FASTA sequences of the wild-type and mutant proteins were used for the analysis.

### Tertiary structure modeling and energy minimization:

The PDB (Protein Data Bank) ID: 6WXJ was used as a structural template to generate mutant models (A770P, I775N, and F849S) in PyMol using the “Mutagenesis Wizard.” The most stable rotamers were selected, and models were refined using YASARA (Yet Another Scientific Artificial Reality Application) (http://www.yasara.org/minimizationserver.html), a web-server for protein model refinement [45]. Discovery Studio (DS) visualizer was used for model visualization [46].

### Evaluation of mutant models:

The refined mutant models were evaluated using SAVES v6.0 (https://saves.mbi.ucla.edu/), a collection of six web-based tools for protein structure evaluation. PROCHECK within SAVES was used to generate Ramachandran plots, which were examined for allowed conformational space [47].

### RMSD value calculation:

TM-Align (https://seq2fun.dcmb.med.umich.edu//TM-align/) was used to compare the wild-type and mutant models. RMSD (Root Mean Square Deviation) and TM-scores (Template Model-Score) were computed to assess global structural deviation. TM-scores range from 0–1 (higher indicating better alignment), and smaller RMSD values indicate closer structural similarity [48, 49].

### Molecular dynamics (MD) simulation:

Molecular dynamics simulations were conducted using the GROMACS package via the WebGro server (https://simlab.uams.edu/) [50]. A well-established simulation protocol [51, 52] was followed, involving the simple point charge (SPC) water model in a triclinic periodic box to solvate the system. In addition, the GROMOS96 43a1 force field was applied to optimize the system. Moreover, the system was equilibrated at 300 K and 1.0 bar. Simulations were run for 50 ns, with 1000 frames recorded. To analyze the simulation findings, we calculated the RMSD and root mean square fluctuation (RMSF) of each atom. Additionally, radius of gyration (Rg) and solvent-accessible surface area (SASA) were calculated to investigate the effects of mutation.

## RESULTS

The CSF1R gene SNP data was retrieved from the NCBI SNP database, dbSNP. The entire set was comprised of 780 missense SNPs, 442 synonymous SNPs, and 21,094 intronic SNPs. Given the potential of missense SNPs to impact protein structure and function, we conducted an initial screening using the 780 missense SNPs.

The 780 nsSNPs were subjected to initial screening through six web-based bioinformatics tools. SIFT predicted 51 out of 780 SNPs as deleterious and 77 as tolerated, while the rest were not reported. These 51 deleterious SNPs were further analyzed using PROVEAN and MutPred. PROVEAN predicted 29 SNPs as deleterious, and the remainder were found to be neutral. MutPred predicted 24 SNPs with scores above the threshold, suggesting potential structural disruption or functional loss. Furthermore, FATHMM categorized 44 SNPs as cancerous, PMut predicted 70 as disease-causing, and Missense3D classified 85 SNPs in the damaging category. 

To reduce false positives, a consensus-based approach was implemented to filter out the deleterious, damaging, cancerous, and disease-causing SNPs. The consensus demonstrating the comparative outputs from the six web-based tools is listed in Supplementary Table S1. SNPs predicted as deleterious, damaging, cancerous, and disease-causing by at least five of the six tools were selected for further analysis. A total of seven SNPs (rs1801271, rs121913390, rs281860269, rs281860271, rs281860273, rs281860277, and rs200489778) were identified that follow this filtering criteria, as shown in Table 1. 

**Table 1:** List of deleterious and damaging SNPs predicted by mutation analysis tools

**rs IDs**	**Amino Acid Change**	**SIFT**	**PROVEAN**	**FATHMM**	**PMut**	**MISSENSE 3D**	**MutPred**
**rs1801271**	Y969C	Deleterious	Deleterious	Cancer	Disease	-	Damaging
**rs121913390**	L301S	Deleterious	Deleterious	Cancer	-	Damaging	Damaging
**rs281860269**	E633K	Deleterious	Deleterious	Cancer	-	Damaging	Damaging
**rs281860271**	A770P	Deleterious	Deleterious	Cancer	-	Damaging	Damaging
**rs281860273**	I775N	Deleterious	Deleterious	Cancer	-	Damaging	Damaging
**rs281860277**	F849S	Deleterious	Deleterious	Cancer	-	Damaging	Damaging
**rs200489778**	T663M	Deleterious	Deleterious	Cancer	-	Damaging	Damaging

Following consensus filtering, protein stability analysis was conducted by MuPro, I-Mutant, and iSTABLE algorithms. MuPro and I-Mutant 2.0 are ML-based algorithms, while iSTABLE is based on meta-approach that determines protein stability based on sequence data. Again, a consensus-based approach was used in which the confidence score from iSTABLE and ∆∆G values from the MuPro and I-Mutant were compared (Supplementary Table S2) to filter out those nsSNPs that decrease protein stability**.** We have found that four of the seven nsSNPs (rs121913390, rs281860271, rs281860273, rs281860277) were found to decrease protein stability as shown in Table 2.

**Table 2:** Protein stability predictions by I-Mutant, iSTABLE, and MuPro

**rs IDs**	**AMINO** **ACID MUTATIONS**	**MuPro** **(∆∆G value)**	**I-Mutant** **(∆∆G value)**	**iSTABLE** **(Score)**
**rs121913390**	L301S	-1.8671494 (Decrease)	-3.14 (Decrease)	0.89314 (Decrease)
**rs281860271**	A770P	-1.3311997 (Decrease)	-2.97 (Decrease)	0.781124 (Decrease)
**rs281860273**	I775N	- 2.1269065 (Decrease)	-1.11 (Decrease)	0.857828 (Decrease)
**rs281860277**	F849S	-1.3760635 (Decrease)	-2.53 (Decrease)	0.872933 (Decrease)

The deleterious nsSNPs that predicted to affect protein stability were analysed further using Project HOPE. For the rs ID: rs121913390 (L301S), the mutant amino acid residue (serine) was observed to be smaller than the wild-type residue (lysine). This mutation was found to creates a void in the protein core and to cause a loss of hydrophobic interactions. The mutation was located in the Ig-like C2-type 4 domain. Furthermore, substitution of wild-type alanine residue with the proline residue (rs ID: rs281860271 (A770P)) introduce a larger mutant residue. Since the alanine is located on the protein's surface, this mutation may disrupt interactions with other molecules. The addition of proline can also destabilize the α-helix, potentially leading to significant structural alterations in the protein. This mutation was found to be occurred within the kinase domain of CSF1R. In the case of isoleucine to asparagine substitution (rs281860273 (I775N)), the mutant residue was found to be bigger. The wild-type residue isoleucine was buried within the protein core, and the bulkier asparagine cannot be accommodated in this space, thus resulting in the loss of the hydrophobic interactions. In the instance of rs281860277 (F849S), the mutant residue serine has been observed to be smaller than the wild-type phenylalanine. This mutation causes a loss of hydrophobic interactions in the core of the protein and it also causes a void in the core of the protein (Table 3).

**Table 3:** Summary of structural consequences observed for each mutation using Project HOPE, highlighting alterations in amino acid size, hydrophobicity, and spatial accommodation

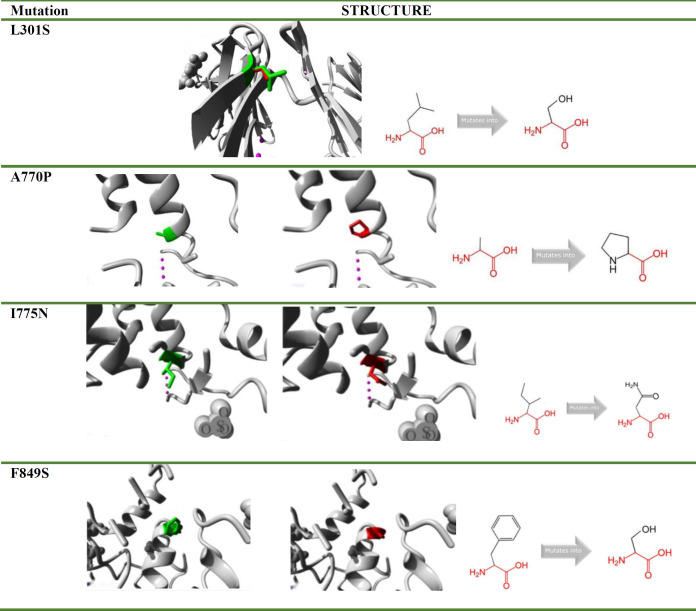

Secondary structure prediction was performed using PSIPRED. The FASTA sequences both wild-type and mutant proteins were submitted as inputs, with mutations manually inserted. In L301S (rs121913390), the loss of beta-strands at positions 48-50, 341-342, 350-352, 436-437, 471-474, and 953-955 was predicted (Fig. S1). Moreover, the new strand was found to be introduced at positions 358-360, 562-63,575-577,724-725 and 775-776 (Fig. S1B). Beside this, a helix was detected at position 701–702, and a helix-to-strand transition was observed at position 168–170 (Fig. S1B).

The PSIPRED analysis of A770P revealed strand additions at 6-7, 22-23, and 576-577, and deletions at 48-50 and 953-955 (Fig. S1C). A strand at 618-619 was replaced by a helix. Strands were lost at 456-457, 784-786, and 797-802, while new helices emerged at 573-574, 699-700, and 702-704 (Fig. S1C). For I775N, strands were found at positions 23-24, 109-110, 576-577, 775-777, and 791-794 (Fig. S1D), and a helix was predicted at 618-619. In F849S, strand deletion was observed at 953-955, with helix formation at 700-702, and strand introduction at 576-577 (Fig. S1E). These variations were compared with the native structure (Table 4).

**Table 4:** The change in number of strands and helices between the mutants and the wild-type (native state) structure of CSF1R, as predicted by PSIPRED

**Amino acid change**	**No. of helix**	**No. of strands**
**Native State**	20	53
**L301S**	21	54
**A770P**	22	55
**I775N**	21	58
**F849S**	21	56

**Figure 2:**
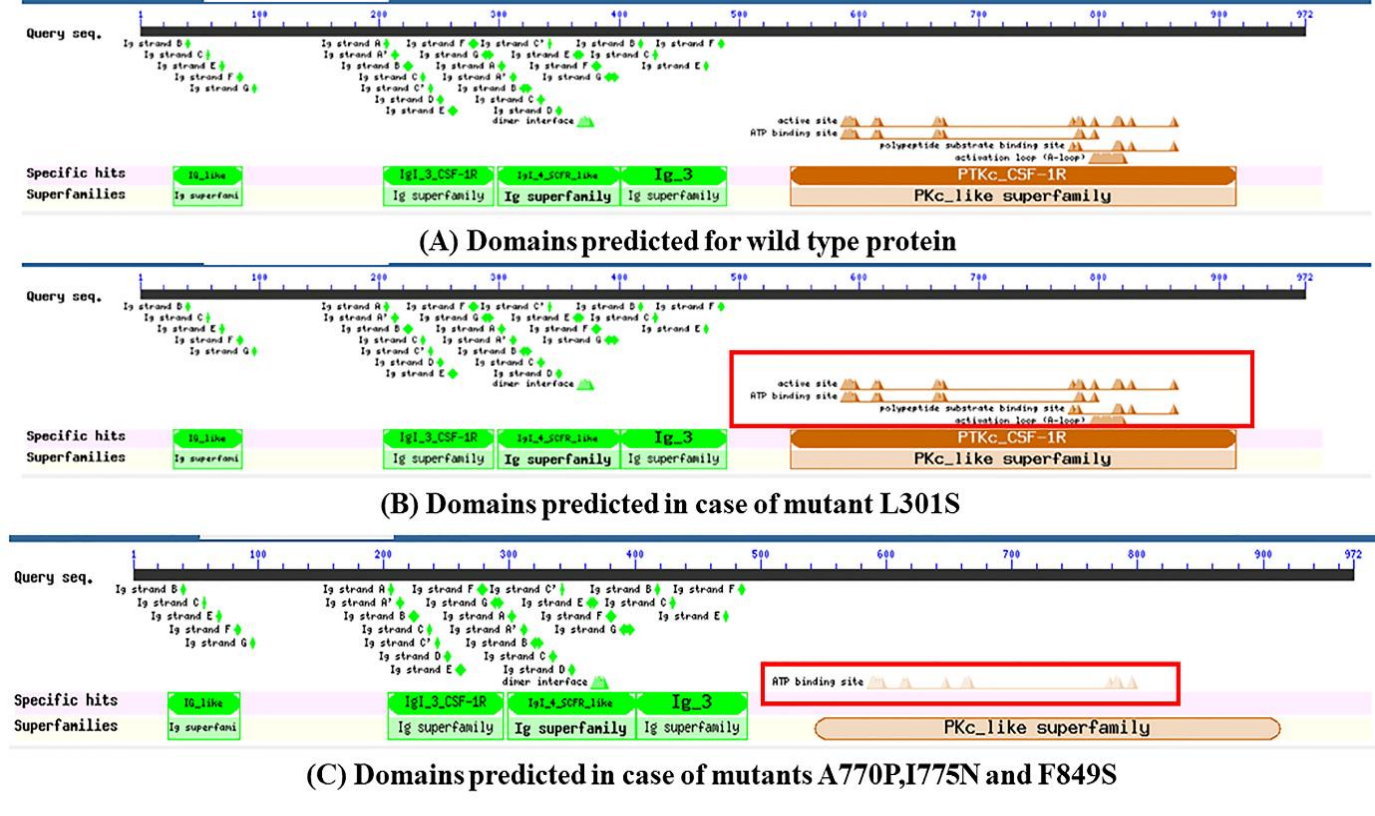
Domains predicted by CDD showed the loss of the active site, polypeptide substrate binding site, and the activation loop (marked in the red box) in mutants A770P, I775N, and F849S **(C)** compared to wild-type **(A)** while mutant L301S **(B)** did not show any loss of these signature elements (marked in the red box).

Domain architecture analysis via CDD indicated the presence of four domains in the wild-type protein: Ig-like domain, Ig3-CSF1R-like domain, Ig-3 domain, and PTKc-CSF1R domain. The PTKc_CSF1R domain contained active-site, ATP-binding, substrate-binding, and activation loop sites. In the A770P, I775N, and F849S mutants, the loss of active, substrate-binding, and activation loop regions was noted (Fig. 2). Surprisingly, no change in domain architecture was observed for the mutant L301S.

Conservation analysis was conducted using ConSurf. Mutation A770P was found in a moderately conserved region (score 8), and I775N and F849S were located in highly conserved regions (score 9) (Fig. S2). These findings suggest that structural and functional alterations may result from mutations in evolutionarily conserved residues. Table 5 lists the mutations occurring in regions with conservation scores from 7 to 9.

**Table 5:** Conservational score for the amino acid positions as predicted by ConSurf

**rs ID**	**AMINO ACID CHANGE**	**CONSURF SCORE**
**rs281860271**	A770P	8/conserved
**rs281860273**	I775N	9/conserved
**rs281860277**	F849S	9/conserved

3D structure prediction was carried out using PyMol. Mutagenesis was performed using the Mutagenesis Wizard. The 6WXJ template was used to model A770P, I775N, and F849S 3D strcutures. These mutants were selected for modeling due to their loss of domain architecture and the alignment of their sequence coordinates within a single PDB template (6WXJ). The L301S mutation was excluded due to domain preservation and a different template. During modeling of the 3D structure of mutants, the water molecules and the heteroatoms present in the template were removed. Multiple rotamers were generated for each mutation, and the one with the highest confidence was selected for structure modeling. 

**Figure 3:**
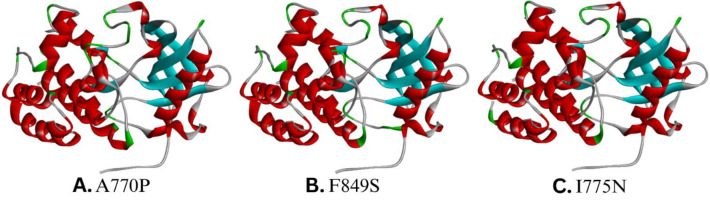
The predicted and energy-refined mutant models generated by PyMOL and YASARA, respectively were visualized using Discovery Studio Visualiser.

**Figure 4:**
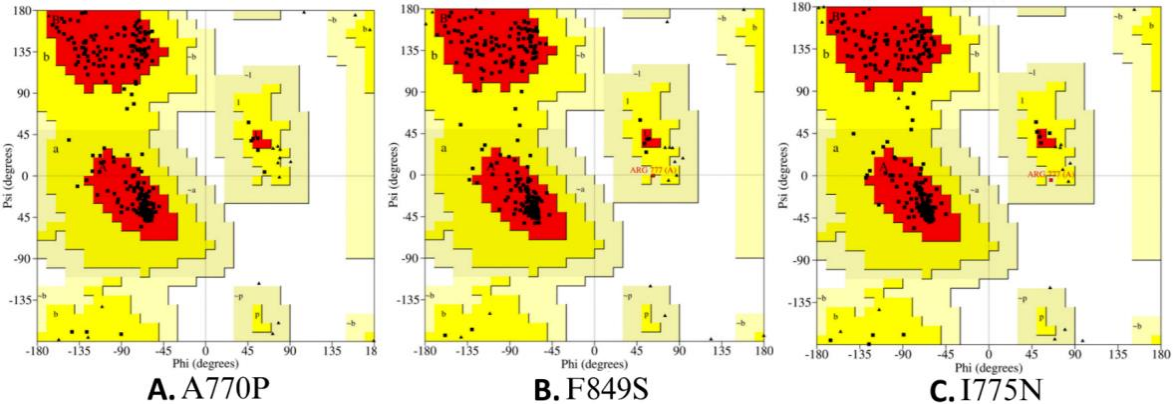
Ramachandran Plots of the mutant models, **(A) **(A770P), **(B)** (F849S) and **(C)** (I775N) generated by PROCHECK. The plots show the clustering of amino acids in the allowed quadrant of the plot.

Furthermore, the geometry optimization of the 3D mutant models was performed by energy minimizations through the YASARA server. Mutant and energy-refined models are shown in Figure 3. Model evaluation was conducted using SAVES v6.0, with PROCHECK used for quality assessment via Ramachandran plot (Fig. 4). The percentage of residues in allowed regions is reported in Table 6.

**Table 6:** Percentage of residues in the allowed regions for three mutants

**rs ID**	**Amino acid change**	**Residues in allowed regions**
**rs281860271**	A770P	91.8%
**rs281860273**	I775N	92.7%
**rs281860277**	F849S	94.3%

The energy-minimized mutant models were analysed using TM-Align for RMSD and TM-score. The TM-Score obtained describes the topological similarity, and the RMSD value determines the deviation of the mutant model from the wild-type protein. Structural similarity and deviation were determined by comparing mutants to the wild-type. Table 7 depicts the values obtained from TM-Align.

**Table 7:** The TM-Scores and the RMSD values of mutant models compared to the wild-type template predicted by TM-Align

**rs ID**	**Amino acid change**	**TM-SCORE**	**RMSD value**
**rs281860271**	A770P	0.99623	0.38
**rs281860273**	I775N	0.99728	0.32
**rs281860277**	F849S	0.99623	0.36

**Figure 5:**
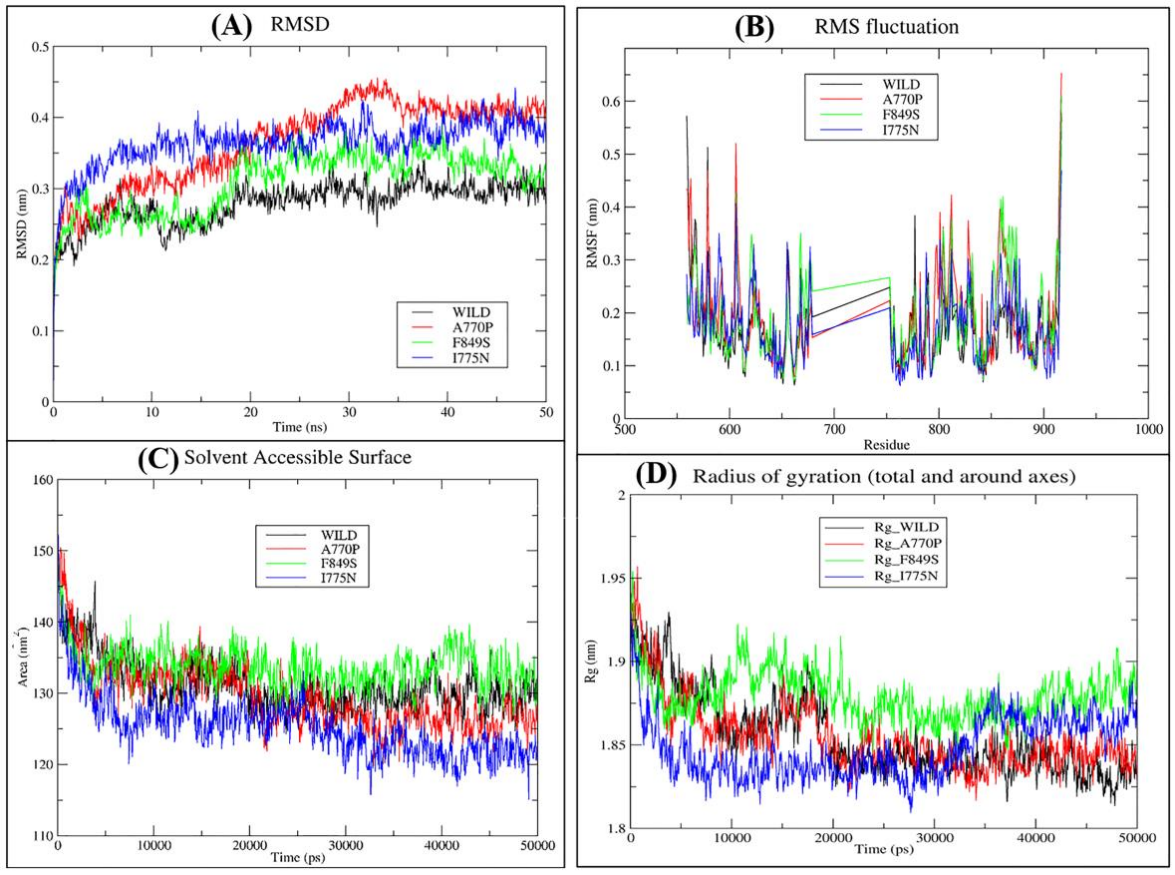
The RMSD **(A)**, RMSF **(B)**, SASA **(C)** and Radius of gyration **(D)** plots of the mutant (A770P, F849S, and I775N) and wild-type protein models obtained by GROMACS simulation package, accessible through the WebGro server.

MD simulations were conducted to examine the initial configurations of mutant protiens for structural flexibility, stability, hydrogen bonding, and solvation over 50 ns in a triclinic box. RMSD was computed to analyze conformational changes. The average RMSD of the native structure was 0.28 nm, while mutants A770P, F849S, and I775N showed increased values of 0.36 nm, 0.31 nm, and 0.36 nm, respectively (Fig. 5A).

To assess dynamic changes, RMSF values were computed. We have observed highest RMSF (0.5773 nm) for ASP917 in the native protein. However, mutant A770P and F849S showed increased RMSF values of 0.653 nm and 0.61 nm, respectively. Surprisingly mutant I775N had the least RMSF value of 0.4683 nm at the same position. Overall, the total RMSF value of the mutant A770P and F849S differed considerably from the native, while mutant I775N showed a similar level of flexibility compared to the native (Fig. 5B).

Protein stability was further assessed by analysing total hydrogen bonds. Notably, the mutants A770P and F849S displayed fewer overall hydrogen bonds. Furthermore, we calculated the SASA values for both the native and mutants. It is worth noting that the F849S mutant exhibited a considerably higher average SASA value of 133.67 nm^2^ in contrast to the native protein (131 nm^2^). Conversely, I775N and A770P showed lower average SASA values of 129.61 nm^2^ and 125.22 nm^2^, respectively, compared to native (Fig. 5C). The Rg was evaluated to assess compactness. The native average Rg value was recorded as 1.85 nm, while the values for I775N, F849S, and A770P were found to be 1.84 nm, 1.87 nm, and 1.85 nm, respectively. F849S displayed the greatest Rg fluctuation (Fig. 5D).

## DISCUSSION

The emergence of high-throughput genome sequencing technology has made it possible to identify a large number of SNPs. This emphasizes the necessity of thorough investigations to evaluate the clinical significance of these SNPs [53, 54]. Furthermore, in the advancing age of precision medicine, analyzing vast amounts of SNP data can provide valuable insights into the structural and functional variations in gene products that can impact several physiological and pathological processes [55, 56]. 

Moreover, in the last few years, various studies have determined the effect of CSF1R SNPs on various pathophysiological conditions [57-61]. Still, given the rise of SNP data, there is an increasing need and scope for more analysis. Thus, the present study adds to this objective by comprehensively evaluating CSF1R SNPs and elucidating their potential influence on protein structure and function. The study used a combination of bioinformatics and molecular dynamics simulation tools to predict the high-risk deleterious missense SNPs. In this work, we analyzed a large dataset of 780 missense SNPs of the *CSF1R* gene to identify deleterious SNPs. A consensus-based approach was followed, which included selecting those SNPs that were predicted deleterious or disease-causing across at least five of the six tools used. A consensus-based approach minimized the chances of false-positive predictions, as demonstrated by numerous prior studies that used multiple tool consensuses to enhance predictive accuracy [62-65]. We have identified seven missense SNPs (Y969C, L301S, E633K, A770P, I775N, F849S, and T663M) in the category of deleterious, damaging or disease-causing by at least five tools. These filtered SNPs were considered for further downstream analysis.

Predicting changes in protein stability due to polymorphism in genetic data is of great significance because it impacts personalized medicine and diagnostics. With the increased sensitivity and specificity of the ML-based tools for predicting protein stability upon point mutations, a large volume of SNP data has been screened [62, 66, 67]. In our analysis, we found four of the seven identified SNPs (rs121913390 (L301S), rs281860271 (A770P), rs281860273 (I775N), and rs281860277 (F849S)) were predicted to decrease protein stability, as indicated by the prediction results of MuPro, I-Mutant, and iSTABLE. This finding is crucial because reduced protein stability is often found to be associated with changes in protein functions; for instance, a work by Gerasimavicius et al. deciphered the different roles of loss-of-function, gain-of-function, and dominant-negative mutations on the protein [68]. 

In addition, the structural analyses performed using Project HOPE and PSIPRED provided more insights into the molecular mechanisms that underlie the impacts of these SNPs. The L301S and F849S mutations have been predicted to create voids inside the protein core due to the substitution of larger wild-type residues with smaller mutant residues. These voids may likely disrupt the hydrophobic core, resulting in an overall decrease in structural integrity. 

Previous research had also shown a similar pattern in the Fibroblast Growth Factor Receptor 1 (*FGFR1*) gene, resulting in a decrease in stability upon substitution (P722S) of larger wild-type residues with smaller mutant residues [69, 70]. Besides this, the A770P mutation would be predicted to hinder the protein interactions on the surface, thus possibly affecting the protein's ability to interact with other molecules or substrates. In contrast, the I775N mutation resulted in the insertion of a bulkier residue within the protein core, which could lead to steric clashes and further destabilization of the protein structure. These findings are consistent with previously published work that has identified steric hindrance as a key factor in protein destabilization due to SNPs [71].

 The secondary structure predictions by PSIPRED revealed considerable alterations in the protein's secondary structure elements due to these mutations. For example, the mutants exhibit beta-strand and alpha-helices changes, indicating that these SNPs cause substantial conformational changes, which could alter the protein's functional domains. The secondary structure analysis of the A770P mutant revealed an unexpected increase in both beta-strands and alpha-helices. Although proline is classically known to disrupt alpha-helices, the A770P mutation showed an increase in both strands and helices. This apparent contradiction may be due to compensatory structural rearrangements, where regions adjacent to the disrupted site reconfigure into stable secondary elements. Moreover, some coil regions may have adopted more ordered structures as a consequence of local conformational stress redistribution induced by the proline substitution. Such behavior, though less common, has been documented in previous studies involving proline mutations [72]. These findings are especially significant considering the role of CSF1R protein in signaling pathways related to cell proliferation and differentiation..

Moreover, the domain analysis also found that mutations such as A770P, I775N, and F849S resulted in the loss of critical functional sites, including the active and ATP binding sites. The structural and functional aberrations noted in this work could lead to loss of CSF1R activities which can result in the lack of microglia and disturbance in brain development that were observed by Erblich et al. in their study on homozygous mouse bearing null mutation of *CSF1R* gene [73]. 

During the evolutionary conservation analysis, these mutations were found to occur within highly conserved regions of the protein, underscoring their potential to disrupt essential functions. The high conservation scores of 8 (A770P) and 9 (I775N, F849S) associated with these SNPs suggest that these residues are critical for maintaining the structural and functional integrity of CSF1R, and mutations in these are expected to have deleterious effects. The profound impact of SNPs in modifying evolutionary conservation regions of genes has been a subject of tremendous significance that helps in understanding the structural and functional changes occurring over time [74-79]. 

Lastly, the molecular dynamics simulations validated the destabilizing effects of the identified deleterious SNPs. The higher RMSD for the mutant structures than the native structure indicates that these SNPs lead to greater conformational flexibility and instability. Specifically, the mutations A770P and I775N showed the highest RMSD values, indicating significant deviations from the wild-type protein. Furthermore, the RMSF analysis supported these findings, with mutations A770P and F849S causing increased flexibility at the residue level, notably at position ASP917. Interestingly, the I775N mutation displayed a lower RMSF value at this position, indicating a complex interplay between local flexibility and overall protein stability. 

The present study comprehensively screened the deleterious nsSNPs based on functional and structural stability analysis. However, due to its dependance on computational methods, it prompted future research that should aim to decipher the biological implications of these mutations using functional in-vitro and in-vivo assays. 

In conclusion, this study provides an in-depth analysis of the functional and structural impacts of deleterious nsSNPs in the *CSF1R* gene. By employing a consensus-based in-silico approach, four highly deleterious mutations, such as L301S, A770P, I775N, and F849S, were identified. Furthermore, structural analysis revealed that these mutations not only destabilize the protein, particularly within conserved regions, but also lead to substantial alterations in secondary structure and domain architecture. Specifically, the A770P, I775N, and F849S mutations resulted in the gain or loss of beta-strands and alpha-helices and the disruption of essential functional domains such as active sites and ATP-binding regions. Moreover, MD simulations highlighted increased conformational flexibility and instability in the mutated proteins. Further in vitro and in vivo instigations are required to validate these predictions and explore therapeutic interventions targeting these mutations, potentially contributing to the development of treatments for diseases associated with CSF1R dysfunction.
