# Supplementary material for: A systematic in-silico functional and structural analysis reveals deleterious missense nsSNPs in the human CSF1R gene
Source: Mol Biol Res Commun. 2025;14(4):291–306. doi: 10.22099/mbrc.2025.53206.2156 (PMC12426959; doi:10.22099/mbrc.2025.53206.2156)
Supplement: Supplementary file 1 — Supplementary Figures S1-S2 [file mbrc-14-291-s001.pdf]

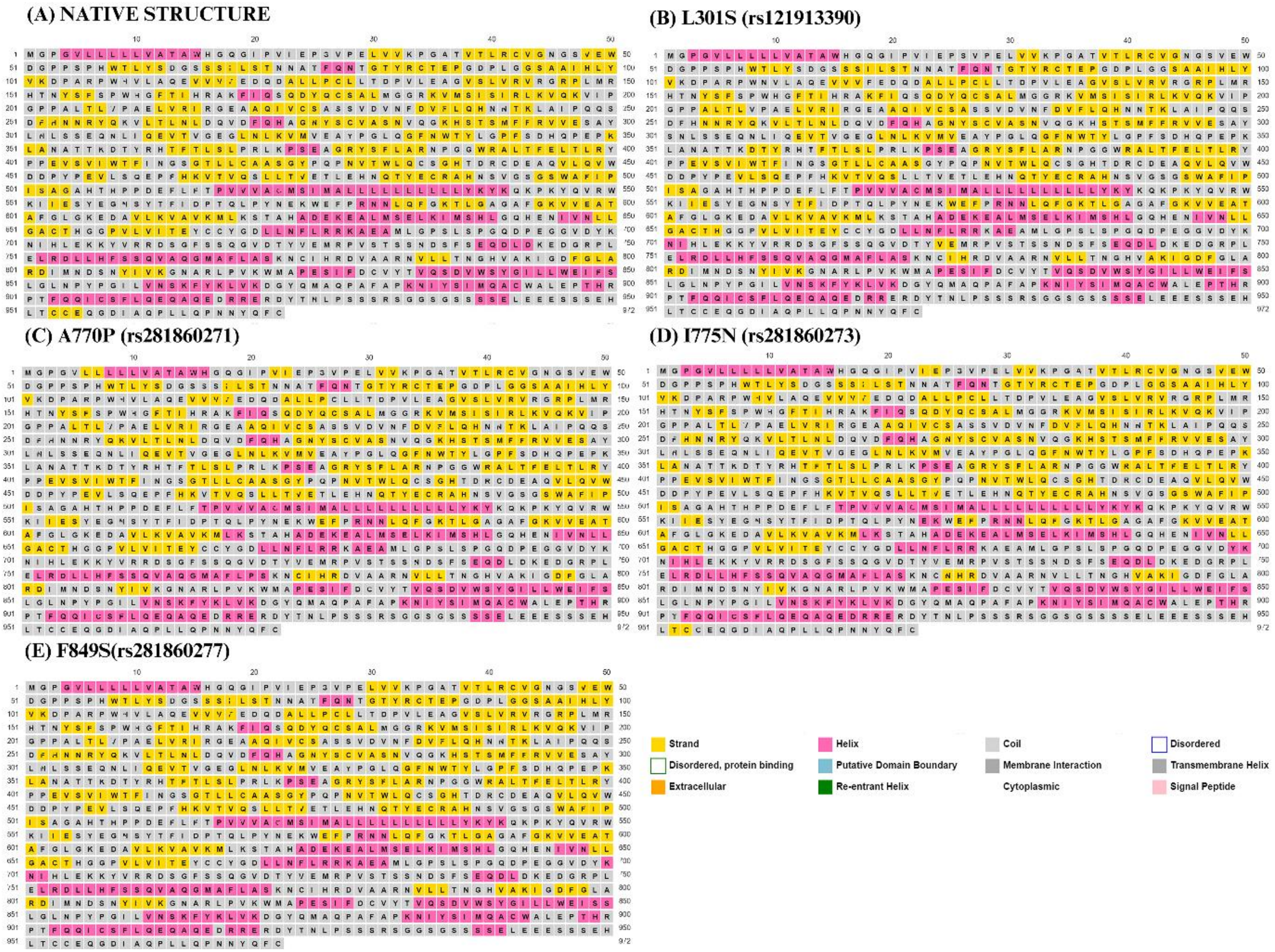

Figure S1: The alteration in secondary structures of native (A) and the mutants (B-E) predicted by PSIPRED. Different secondary structure types such as strand, helix, and coil etc. were shown along the sequence coordinates.

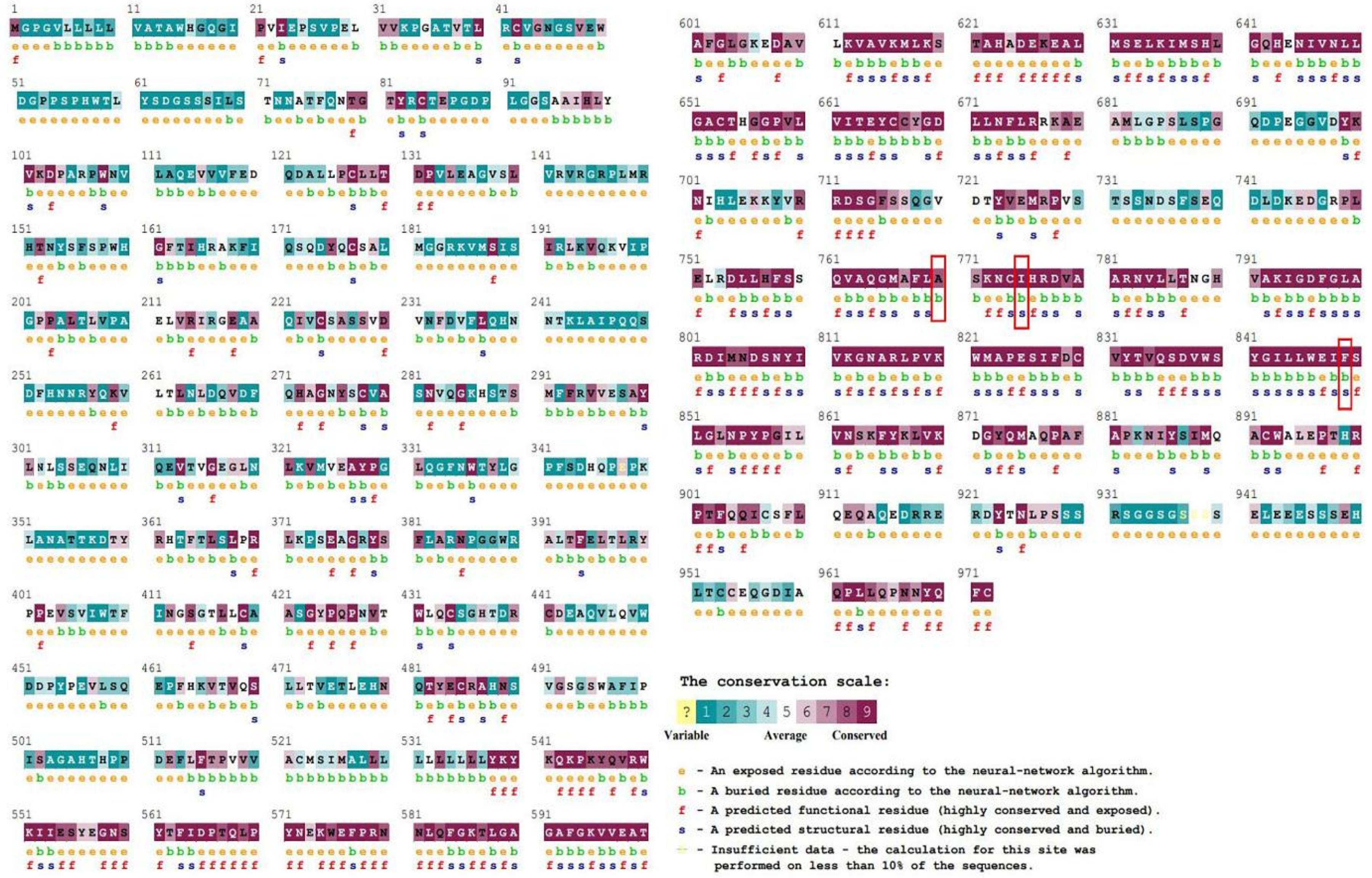

Figure S2: ConSurf server predicted the conservational score of each residue of the wild-type CSF1R. Scores for the three mutation positions were predicted to be 8 and 9 (marked by the red box), such high scores represented highly conserved regions.

Table S1: The comparative outputs from the six web-based tools

rs ID's	Amino acid change	SIFT	PROVEAN	FATHMM	Pmut		Missense 3D	MutPred	PERCENTAGE
rs1801271	Y969F	DELETERIOUS	NEUTRAL	CANCER	**0.54 (80%)**	Disease	NEUTRAL	-	3 of 6
rs1801271	Y969C	DELETERIOUS	Deleterious	CANCER	**0.68 (85%)**	Disease	NEUTRAL	0.812	5 of 6
rs56048668	V32G	DELETERIOUS	Deleterious	NEUTRAL	**0.86 (91%)**	Disease	NEUTRAL	0.822	4 of 6
rs121913390	L301S	DELETERIOUS	Deleterious	CANCER	**0.26 (90%)**	Neutral	Damaging	0.559	5 of 6
rs121913393	Y969H	DELETERIOUS	NEUTRAL	CANCER	**0.62 (83%)**	Disease	NEUTRAL	0.737	4 of 6
rs138432536	V518M	DELETERIOUS	NEUTRAL	CANCER	**0.26 (90%)**	Neutral	NEUTRAL	0.541	3 of 6
rs140924076	A123G	DELETERIOUS	Deleterious	NEUTRAL	**0.43 (85%)**	Neutral	NEUTRAL	-	2 of 6
rs143025739	R106W	DELETERIOUS	Deleterious	NEUTRAL	**0.81 (89%)**	Disease	NEUTRAL	-	3 of 6
rs143274491	V268I	DELETERIOUS	NEUTRAL	NEUTRAL	-		NEUTRAL	-	1 of 6
rs146406037	N255I	DELETERIOUS (LOW CONFIDENCE)	NEUTRAL	NEUTRAL	-		NEUTRAL	-	1 of 6
rs150475750	P90T	DELETERIOUS	NEUTRAL	NEUTRAL	-		NEUTRAL	-	1 of 6
rs200788902	T600M	DELETERIOUS	Deleterious	CANCER	-		NEUTRAL	-	3 of 6
rs202216061	R921W	DELETERIOUS	NEUTRAL	CANCER	**0.70 (86%)**	Disease	NEUTRAL	-	3 of 6
rs281860268	G589E	DELETERIOUS	Deleterious	CANCER	**0.26 (90%)**	Neutral	NEUTRAL	0.916	4 of 6
rs281860269	E633K	DELETERIOUS	Deleterious	CANCER	**0.26 (90%)**	Neutral	Damaging	0.766	5 of 6
rs281860270	M766T	DELETERIOUS	Deleterious	CANCER	**0.26 (90%)**	Neutral	NEUTRAL	0.847	4 of 6
rs281860271	A770P	DELETERIOUS	Deleterious	CANCER	**0.26 (90%)**	Neutral	Damaging	0.827	5 of 6
rs281860273	I775N	DELETERIOUS	Deleterious	CANCER	**0.26 (90%)**	Neutral	Damaging	0.932	5 of 6
rs281860274	I794T	DELETERIOUS	Deleterious	CANCER	**0.26 (90%)**	Neutral	NEUTRAL	0.85	4 of 6
rs281860277	F849S	DELETERIOUS	Deleterious	CANCER	**0.26 (90%)**	Neutral	Damaging	0.893	5 of 6
rs281860278	L868P	DELETERIOUS	Deleterious	CANCER	**0.26 (90%)**	Neutral	NEUTRAL	0.746	4 of 6
rs281860279	M875T	DELETERIOUS	Deleterious	CANCER	**0.26 (90%)**	Neutral	NEUTRAL	0.899	4 of 6

rs281860280	P878T	DELETERIOUS	Deleterious	CANCER	**0.26 (90%)** Neutral	NEUTRAL	0.861	4 of 6
rs281860281	R782H	DELETERIOUS	Deleterious	CANCER	**0.26 (90%)** Neutral	NEUTRAL	0.903	4 of 6
rs376495524	R214Q	DELETERIOUS (WARNING LOW CONFIDENCE)	NEUTRAL	NEUTRAL	**0.26 (90%)** Neutral	NEUTRAL	-	1 of 6
rs387906662	D837Y	DELETERIOUS	Deleterious	NEUTRAL	**0.26 (90%)** Neutral	Damaging	0.927	4 of 6
rs17855673	P247H	DELETERIOUS (WARNING LOW CONFIDENCE)	NEUTRAL	NEUTRAL	**0.26 (90%)** Neutral	NEUTRAL	-	1 of 6
rs41529644	T567M	DELETERIOUS	Deleterious	CANCER	**0.26 (90%)** Neutral	NEUTRAL	-	3 of 6
rs139635308	T37M	DELETERIOUS	NEUTRAL	NEUTRAL	**0.29 (89%)** Neutral	NEUTRAL	-	1 of 6
rs139797829	K867Q	DELETERIOUS	Deleterious	CANCER	**0.26 (90%)** Neutral	NEUTRAL	-	3 of 6
rs141621829	D63H	DELETERIOUS	NEUTRAL	NEUTRAL	**0.26 (90%)** Neutral	NEUTRAL	-	1 of 6
rs142978503	Y723H	DELETERIOUS	Deleterious	CANCER	**0.26 (90%)** Neutral	NEUTRAL	-	3 of 6
rs143941779	R184T	DELETERIOUS	NEUTRAL	NEUTRAL	**0.26 (90%)** Neutral	NEUTRAL	-	1 of 6
rs144261133	G52V	DELETERIOUS	NEUTRAL	NEUTRAL	**0.21 (92%)** Neutral	NEUTRAL	-	1 of 6
rs144414073	P927L	DELETERIOUS	Deleterious	CANCER	**0.48 (83%)** Neutral	NEUTRAL	-	3 of 6
rs147811334	R142C	DELETERIOUS	NEUTRAL	NEUTRAL	**0.70 (86%)** Disease	NEUTRAL	-	2 of 6
rs148727692	E29K	DELETERIOUS	NEUTRAL	NEUTRAL	**0.74 (87%)** Disease	NEUTRAL	-	2 of 6
rs149753251	S414F	DELETERIOUS	NEUTRAL	CANCER	**0.26 (90%)** Neutral	NEUTRAL	0.556	3 of 6
rs200068110	E955K	DELETERIOUS	NEUTRAL	CANCER	-	NEUTRAL	-	1 of 6
rs200489778	T663M	DELETERIOUS	Deleterious	CANCER	**0.26 (90%)** Neutral	Damaging	0.687	5 of 6
rs201012043	T516I	DELETERIOUS	Deleterious	CANCER	**0.26 (90%)** Neutral	NEUTRAL	-	3 of 6
rs368661705	L683P	DELETERIOUS	Deleterious	NEUTRAL	**0.26 (90%)** Neutral	Damaging	0.581	4 of 6
rs369879651	E745Q	DELETERIOUS	NEUTRAL	NEUTRAL	**0.26 (90%)** Neutral	NEUTRAL	-	1 of 6
rs370585355	L514H	DELETERIOUS	Deleterious	NEUTRAL	**0.26 (90%)** Neutral	NEUTRAL	-	2 of 6
rs370624765	R549C	DELETERIOUS	Deleterious	NEUTRAL	**0.26 (90%)** Neutral	NEUTRAL	-	2 of 6
rs370659554	Y561C	DELETERIOUS	Deleterious	NEUTRAL	**0.26 (90%)** Neutral	NEUTRAL	0.69	3 of 6
rs372210450	V32M	DELETERIOUS	NEUTRAL	NEUTRAL	**0.43 (85%)** Neutral	NEUTRAL	0.576	2 of 6

rs374166176	R150C	DELETERIOUS	Deleterious	NEUTRAL	**0.62 (83%)** Disease	NEUTRAL	-	3 of 6
rs34030164	E920D	N/A	N/A	CANCER	-	NEUTRAL	-	1 of 6
rs34951517	G413S	N/A	N/A	CANCER	-	NEUTRAL	-	1 of 6
rs41355444	G747R	N/A	N/A	CANCER	**0.26 (90%)** Neutral	NEUTRAL	-	1 of 6
rs55942044	L536V	N/A	N/A	CANCER	**0.26 (90%)** Neutral	NEUTRAL	0.503	2 of 6
rs142435467	E916K	N/A	N/A	CANCER	**0.34 (88%)** Neutral	NEUTRAL	-	1 of 6
rs146386843	T587I	N/A	N/A	CANCER	**0.26 (90%)** Neutral	NEUTRAL	-	1 of 6
rs199915312	E739D	N/A	N/A	CANCER	**0.26 (90%)** Neutral	NEUTRAL	-	1 of 6
rs201569135	R710H	N/A	N/A	CANCER	**0.26 (90%)** Neutral	NEUTRAL	-	1 of 6
rs17854478	A629S	N/A	N/A	CANCER	**0.26 (90%)** Neutral	NEUTRAL	-	1 of 6
rs111943087	H703Y	N/A	N/A	CANCER	**0.26 (90%)** Neutral	NEUTRAL	-	1 of 6
rs139061724	V729I	N/A	N/A	CANCER	**0.26 (90%)** Neutral	NEUTRAL	-	1 of 6
rs139203165	P510A	N/A	N/A	CANCER	**0.26 (90%)** Neutral	NEUTRAL	-	1 of 6
rs140118369	S935G	N/A	N/A	CANCER	**0.12 (95%)** Neutral	NEUTRAL	-	1 of 6
rs141866247	G690S	N/A	N/A	CANCER	**0.26 (90%)** Neutral	NEUTRAL	-	1 of 6
rs142543645	H506P	N/A	N/A	CANCER	**0.26 (90%)** Neutral	NEUTRAL	-	1 of 6
rs150668652	G684E	N/A	N/A	CANCER	**0.26 (90%)** Neutral	NEUTRAL	-	1 of 6
rs200284956	I848V	N/A	N/A	CANCER	**0.26 (90%)** Neutral	NEUTRAL	-	1 of 6
rs200925061	E920K	N/A	N/A	CANCER	**0.24 (91%)** Neutral	NEUTRAL	-	1 of 6
rs1230928599	F381L	N/A	N/A	N/A	**0.26 (90%)** Neutral	NEUTRAL	0.706	1 of 6
rs577425911	L7R	N/A	N/A	N/A	**0.57 (81%)** Disease	NEUTRAL	-	1 of 6
rs757795589	P21S	N/A	N/A	N/A	**0.69 (86%)** Disease	NEUTRAL	-	1 of 6
rs757795589	P21T	N/A	N/A	N/A	**0.63 (83%)** Disease	NEUTRAL	-	1 of 6
rs749946644	I23T	N/A	N/A	N/A	**0.82 (90%)** Disease	NEUTRAL	-	1 of 6
COSM1435148	V31A	N/A	N/A	N/A	**0.70 (86%)** Disease	NEUTRAL	-	1 of 6
rs56048668	V32A	N/A	N/A	N/A	**0.71 (86%)** Disease	NEUTRAL	-	1 of 6
rs1397361181	G35A	N/A	N/A	N/A	**0.70 (86%)** Disease	Damaging	-	2 of 6
rs1331779557	G35R	N/A	N/A	N/A	**0.71 (86%)** Disease	Damaging	-	2 of 6
rs1221129051	C42R	N/A	N/A	N/A	**0.93 (94%)** Disease	Damaging	-	2 of 6
rs147408195	G44S	N/A	N/A	N/A	**0.58 (82%)** Disease	NEUTRAL	-	1 of 6

rs756809440	W50C	N/A	N/A	N/A	**0.89 (92%)** Disease	Damaging	-	1 of 6
rs1561940695	W50L	N/A	N/A	N/A	**0.87 (91%)** Disease	NEUTRAL	-	1 of 6
NCI-TCGA novel	W50R	N/A	N/A	N/A	**0.93 (94%)** Disease	NEUTRAL	-	1 of 6
rs777789969	P53L	N/A	N/A	N/A	**0.70 (86%)** Disease	NEUTRAL	-	1 of 6
rs1433299442	P53S	N/A	N/A	N/A	**0.62 (83%)** Disease	NEUTRAL	-	1 of 6
rs1259778590	T71N	N/A	N/A	N/A	**0.57 (81%)** Disease	NEUTRAL	-	1 of 6
rs770380006	T71S	N/A	N/A	N/A	**0.56 (81%)** Disease	NEUTRAL	-	1 of 6
rs1351811725	N73D	N/A	N/A	N/A	**0.58 (82%)** Disease	NEUTRAL	-	1 of 6
rs1325333723	T75A	N/A	N/A	N/A	**0.71 (86%)** Disease	NEUTRAL	-	1 of 6
rs1014062385	T79A	N/A	N/A	N/A	**0.62 (83%)** Disease	Damaging	-	2 of 6
NCI-TCGA novel	T79P	N/A	N/A	N/A	**0.86 (91%)** Disease	Damaging	-	2 of 6
rs1414323194	T79R	N/A	N/A	N/A	**0.82 (90%)** Disease	Damaging	-	2 of 6
rs1014062385	T79S	N/A	N/A	N/A	**0.63 (84%)** Disease	NEUTRAL	-	1 of 6
NCI-TCGA novel	Y82L	N/A	N/A	N/A	**0.88 (92%)** Disease	NEUTRAL	-	1 of 6
rs1391078471	I97N	N/A	N/A	N/A	**0.76 (88%)** Disease	Damaging	-	2 of 6

rs1413974974	Y100C	N/A	N/A	N/A	**0.93 (94%)** Disease	NEUTRAL	-	1 of 6
rs1284272648	Y100H	N/A	N/A	N/A	**0.72 (86%)** Disease	Damaging	-	2 of 6
rs1561939495	D103E	N/A	N/A	N/A	**0.77 (88%)** Disease	NEUTRAL	-	1 of 6
rs971881879	P104R	N/A	N/A	N/A	**0.77 (88%)** Disease	NEUTRAL	-	1 of 6
COSM3852726	P104T	N/A	N/A	N/A	**0.59 (82%)** Disease	NEUTRAL	-	1 of 6
COSM1435140	E119K	N/A	N/A	N/A	**0.51 (79%)** Disease	NEUTRAL	-	1 of 6
rs1561939362	E119Q	N/A	N/A	N/A	**0.69 (86%)** Disease	NEUTRAL	-	1 of 6
rs1244467205	L125M	N/A	N/A	N/A	**0.59 (82%)** Disease	NEUTRAL	-	1 of 6
rs773417438	D131E	N/A	N/A	N/A	**0.58 (82%)** Disease	NEUTRAL	-	1 of 6
rs769905192	P132A	N/A	N/A	N/A	**0.78 (88%)** Disease	NEUTRAL	-	1 of 6
VAR_083140	P132L	N/A	N/A	N/A	**0.87 (91%)** Disease	NEUTRAL	-	1 of 6
NCI-TCGA novel	P132Q	N/A	N/A	N/A	**0.85 (91%)** Disease	NEUTRAL	-	1 of 6
COSM6170008	V138D	N/A	N/A	N/A	**0.90 (93%)** Disease	NEUTRAL	-	1 of 6
rs755093010	V138I	N/A	N/A	N/A	**0.51 (79%)** Disease	NEUTRAL	-	1 of 6
rs569441099	S139L	N/A	N/A	N/A	**0.71 (86%)** Disease	NEUTRAL	-	1 of 6
rs1214590308	R144C	N/A	N/A	N/A	**0.69 (85%)** Disease	NEUTRAL	-	1 of 6

rs776871942	T163A	N/A	N/A	N/A	**0.64 (84%)** Disease	NEUTRAL	-	1 of 6
rs776871942	T163P	N/A	N/A	N/A	**0.69 (86%)** Disease	NEUTRAL	-	1 of 6
COSM143513 8	I164T	N/A	N/A	N/A	**0.70 (86%)** Disease	NEUTRAL	-	1 of 6
rs1268479936	I888V	N/A	N/A	N/A	**0.53 (80%)** Disease	NEUTRAL	-	1 of 6
RCV00073624 5	A891P	N/A	N/A	N/A	**0.61 (83%)** Disease	NEUTRAL	-	1 of 6
NCI-TCGA novel	C892R	N/A	N/A	N/A	**0.92 (94%)** Disease	Damaging	-	2 of 6
rs1390027952	A894V	N/A	N/A	N/A	**0.72 (86%)** Disease	NEUTRAL	-	1 of 6
RCV00014952 8	P901S	N/A	N/A	N/A	**0.91 (93%)** Disease	NEUTRAL	-	1 of 6
RCV00014953 6	I906T	N/A	N/A	N/A	**0.92 (93%)** Disease	NEUTRAL	-	1 of 6
rs924679612	L926P	N/A	N/A	N/A	**0.77 (88%)** Disease	NEUTRAL	-	1 of 6
rs761835712	G944K	N/A	N/A	N/A	**0.62 (83%)** Disease	NEUTRAL	-	1 of 6
rs768503863	S946R	N/A	N/A	N/A	**0.55 (81%)** Disease	NEUTRAL	-	1 of 6
rs755380615	C953R	N/A	N/A	N/A	**0.81 (89%)** Disease	NEUTRAL	-	1 of 6
rs751974904	C953Y	N/A	N/A	N/A	**0.69 (85%)** Disease	NEUTRAL	-	1 of 6
rs1379972794	C954Y	N/A	N/A	N/A	**0.61 (83%)** Disease	NEUTRAL	-	1 of 6
rs200068110	E955L	N/A	N/A	N/A	**0.69 (85%)** Disease	NEUTRAL	-	1 of 6

RCV000497696	D958V	N/A	N/A	N/A	**0.76 (88%)** Disease	NEUTRAL	-	1 of 6
rs776645465	A960V	N/A	N/A	N/A	**0.61 (83%)** Disease	NEUTRAL	-	1 of 6
COSM3852700	P966L	N/A	N/A	N/A	**0.61 (83%)** Disease	NEUTRAL	-	1 of 6
rs771254626	C972W	N/A	N/A	N/A	**0.70 (86%)** Disease	NEUTRAL	-	1 of 6
rs690016559	C653R	N/A	N/A	N/A	**0.26 (90%)** Neutral	Damaging	-	1 of 6
rs1057520014	L301F	N/A	N/A	N/A	**0.26 (90%)** Neutral	Damaging	-	1 of 6
rs886060257	G436S	N/A	N/A	N/A	**0.26 (90%)** Neutral	Damaging	-	1 of 6
rs690016563	L582P	N/A	N/A	N/A	**0.26 (90%)** Neutral	Damaging	-	1 of 6
rs690016547	L630R	N/A	N/A	N/A	**0.26 (90%)** Neutral	Damaging	-	1 of 6
rs397515555	C653Y	N/A	N/A	N/A	**0.26 (90%)** Neutral	Damaging	-	1 of 6
rs690016566	G765D	N/A	N/A	N/A	**0.26 (90%)** Neutral	Damaging	-	1 of 6
rs397515556	R777W	N/A	N/A	N/A	**0.26 (90%)** Neutral	Damaging	-	1 of 6
rs690016548	R777Q	N/A	N/A	N/A	**0.26 (90%)** Neutral	Damaging	-	1 of 6
rs587777247	A781E	N/A	N/A	N/A	**0.26 (90%)** Neutral	Damaging	-	1 of 6
rs690016561	K793T	N/A	N/A	N/A	**0.26 (90%)** Neutral	Damaging	-	1 of 6
rs690016549	L817P	N/A	N/A	N/A	**0.26 (90%)** Neutral	Damaging	-	1 of 6
rs690016549	L817Q	N/A	N/A	N/A	**0.26 (90%)** Neutral	Damaging	-	1 of 6
rs690016555	I843N	N/A	N/A	N/A	**0.26 (90%)** Neutral	Damaging	-	1 of 6

rs690016552	Y856H	N/A	N/A	N/A	**0.26 (90%)** Neutral	Damaging	-	1 of 6
rs1057519156	V869G	N/A	N/A	N/A	**0.26 (90%)** Neutral	Damaging	-	1 of 6
rs1256984931	V48M	N/A	N/A	N/A	**0.44 (84%)** Neutral	Damaging	-	1 of 6
rs767283490	W58R	N/A	N/A	N/A	**0.22 (92%)** Neutral	Damaging	-	1 of 6
rs1369244827	G64V	N/A	N/A	N/A	**0.35 (88%)** Neutral	Damaging	-	1 of 6
rs1369244827	G64D	N/A	N/A	N/A	**0.31 (89%)** Neutral	Damaging	-	1 of 6
rs1369244827	G64A	N/A	N/A	N/A	**0.10 (96%)** Neutral	Damaging	-	1 of 6
rs140924076	A123E	N/A	N/A	N/A	**0.46 (84%)** Neutral	Damaging	-	1 of 6
rs1456318164	L134P	N/A	N/A	N/A	**0.16 (93%)** Neutral	Damaging	-	1 of 6
rs779121430	S178G	N/A	N/A	N/A	**0.26 (90%)** Neutral	Damaging	-	1 of 6
rs1228085120	S178T	N/A	N/A	N/A	**0.26 (90%)** Neutral	Damaging	-	1 of 6
rs759685769	G201A	N/A	N/A	N/A	**0.26 (90%)** Neutral	Damaging	-	1 of 6
rs1161693014	G201R	N/A	N/A	N/A	**0.26 (90%)** Neutral	Damaging	-	1 of 6
rs201523105	A204P	N/A	N/A	N/A	**0.26 (90%)** Neutral	Damaging	-	1 of 6
rs754385244	L212Q	N/A	N/A	N/A	**0.26 (90%)** Neutral	Damaging	-	1 of 6
rs746530522	V213M	N/A	N/A	N/A	**0.26 (90%)** Neutral	Damaging	-	1 of 6
rs1465872803	V235G	N/A	N/A	N/A	**0.26 (90%)** Neutral	Damaging	-	1 of 6
rs1354335625	L265P	N/A	N/A	N/A	**0.26 (90%)** Neutral	Damaging	-	1 of 6
rs758675858	G274S	N/A	N/A	N/A	**0.26 (90%)** Neutral	Damaging	-	1 of 6
rs928018985	G285S	N/A	N/A	N/A	**0.26 (90%)** Neutral	Damaging	-	1 of 6
rs1221149937	G285D	N/A	N/A	N/A	**0.26 (90%)** Neutral	Damaging	-	1 of 6
rs753056795	S288P	N/A	N/A	N/A	**0.26 (90%)** Neutral	Damaging	-	1 of 6
-	A354P	N/A	N/A	N/A	**0.26 (90%)** Neutral	Damaging	-	1 of 6
rs771375056	Y400H	N/A	N/A	N/A	**0.26 (90%)** Neutral	Damaging	-	1 of 6
rs766613030	S414P	N/A	N/A	N/A	**0.26 (90%)** Neutral	Damaging	-	1 of 6
rs926797716	T416I	N/A	N/A	N/A	**0.26 (90%)** Neutral	Damaging	-	1 of 6
-	G423V	N/A	N/A	N/A	**0.26 (90%)** Neutral	Damaging	-	1 of 6
-	P425L	N/A	N/A	N/A	**0.26 (90%)** Neutral	Damaging	-	1 of 6
-	P425S	N/A	N/A	N/A	**0.26 (90%)** Neutral	Damaging	-	1 of 6
rs773109201	C441S	N/A	N/A	N/A	**0.26 (90%)** Neutral	Damaging	-	1 of 6
rs1322858120	L447P	N/A	N/A	N/A	**0.26 (90%)** Neutral	Damaging	-	1 of 6

rs758434386	P455L	N/A	N/A	N/A	**0.26 (90%)** Neutral	Damaging	-	1 of 6
rs779109656	V466G	N/A	N/A	N/A	**0.26 (90%)** Neutral	Damaging	-	1 of 6
rs773709407	H479P	N/A	N/A	N/A	**0.26 (90%)** Neutral	Damaging	-	1 of 6
rs374872712	Q481R	N/A	N/A	N/A	**0.26 (90%)** Neutral	Damaging	-	1 of 6
rs1452088406	E484Q	N/A	N/A	N/A	**0.26 (90%)** Neutral	Damaging	-	1 of 6
-	C485R	N/A	N/A	N/A	**0.26 (90%)** Neutral	Damaging	-	1 of 6
rs770605687	P544L	N/A	N/A	N/A	**0.26 (90%)** Neutral	Damaging	-	1 of 6
rs1179809363	Q547R	N/A	N/A	N/A	**0.26 (90%)** Neutral	Damaging	-	1 of 6
rs371219353	V548I	N/A	N/A	N/A	**0.26 (90%)** Neutral	Damaging	-	1 of 6
rs1219122867	W550C	N/A	N/A	N/A	**0.26 (90%)** Neutral	Damaging	-	1 of 6
rs184499252	H643P	N/A	N/A	N/A	**0.26 (90%)** Neutral	Damaging	-	1 of 6
-	C653F	N/A	N/A	N/A	**0.26 (90%)** Neutral	Damaging	-	1 of 6
rs1483961692	G657D	N/A	N/A	N/A	**0.26 (90%)** Neutral	Damaging	-	1 of 6
rs777963695	G657R	N/A	N/A	N/A	**0.26 (90%)** Neutral	Damaging	-	1 of 6
rs748228488	G747V	N/A	N/A	N/A	-	Damaging	-	1 of 6
rs1209791630	G798R	N/A	N/A	N/A	**0.26 (90%)** Neutral	Damaging	-	1 of 6
-	A823P	N/A	N/A	N/A	**0.26 (90%)** Neutral	Damaging	-	1 of 6
rs549775847	A914T	N/A	N/A	N/A	**0.11 (95%)** Neutral	Damaging	-	1 of 6
rs549775847	A914P	N/A	N/A	N/A	**0.20 (92%)** Neutral	Damaging	-	1 of 6
rs777093657	A914D	N/A	N/A	N/A	**0.34 (88%)** Neutral	Damaging	-	1 of 6
rs568352098	L303F	N/A	N/A	N/A	**0.26 (90%)** Neutral	Damaging	-	1 of 6

Table S2: Comparison of the confidence score from iSTABLE and ΔΔG values from the MuPro and I-Mutant

rs ID	Amino acid change	I-Mutant DDG VALUE	Protein stability I-Mutant	iSTABLE confidence score	Protein stability iSTABLE	MuPro DDG VALUE	Protein stability MuPro	CONSENSUS
rs121913390	L301S	-3.14	DECREASE	0.89314	Decrease	-1.8671494	Decrease	3 of 3
rs281860271	A770P	-2.97	DECREASE	0.781124	Decrease	-1.3311997	Decrease	3 of 3
rs281860273	I775N	-1.11	DECREASE	0.857828	Decrease	-2.1269065	Decrease	3 of 3
rs281860277	F849S	-2.53	DECREASE	0.872933	Decrease	-1.3760635	Decrease	3 of 3
rs1801271	Y969C	1.01	INCREASE	0.593886	Decrease	-0.58670187	Decrease	2 of 3
rs281860269	E633K	0.01	INCREASE	0.513944	Decrease	-1.4414132	Decrease	2 of 3
rs200489778	T663M	0.14	INCREASE	0.727604	Increase	0.15187933	Increase	0 of 3
